# Pituitary metastasis as the first sign of distant spread in mandibular squamous cell carcinoma

**DOI:** 10.1093/jscr/rjag315

**Published:** 2026-04-28

**Authors:** Angeline A Truong, Jason Chan, Hyunseok Kang, Jia-Shu Chen, Manish Aghi, Patricia Loftus, Mary Jue Xu, Katherine C Wai

**Affiliations:** Department of Otolaryngology–Head and Neck Surgery, University of California, San Francisco, 2233 Post Street, 3rd Floor, San Francisco, CA 94115, United States; Department of Radiation Oncology, University of California, San Francisco, 1600 Divisadero Street, San Francisco, CA 94115, United States; Department of Medicine, University of California, San Francisco, 505 Parnassus Avenue, San Francisco, CA 94143, United States; Department of Neurological Surgery, University of California, San Francisco, 505 Parnassus Avenue, San Francisco, CA 94143, United States; Department of Neurological Surgery, University of California, San Francisco, 505 Parnassus Avenue, San Francisco, CA 94143, United States; Department of Otolaryngology–Head and Neck Surgery, University of California, San Francisco, 2233 Post Street, 3rd Floor, San Francisco, CA 94115, United States; Department of Otolaryngology–Head and Neck Surgery, University of California, San Francisco, 2233 Post Street, 3rd Floor, San Francisco, CA 94115, United States; Department of Otolaryngology–Head and Neck Surgery, University of California, San Francisco, 2233 Post Street, 3rd Floor, San Francisco, CA 94115, United States

**Keywords:** pituitary metastasis, head and neck cancer, squamous cell carcinoma

## Abstract

To describe a rare case of pituitary metastasis arising from mandibular squamous cell carcinoma (SCC), in which pituitary-related symptoms represented the first clinical evidence of distant metastatic disease. Case report and targeted literature review. A patient with previously treated mandibular SCC presented with new-onset visual disturbance and endocrine dysfunction. Imaging, endocrine evaluation, and histopathologic confirmation were performed. Published reports of pituitary metastases from head and neck SCC were reviewed. Magnetic resonance imaging demonstrated an enhancing sellar mass with suprasellar extension and infundibular thickening concerning for metastatic disease. Endoscopic endonasal biopsy confirmed metastatic keratinizing SCC consistent with the primary mandibular tumor. No other distant metastases were identified on staging imaging. Visual field deficits and partial hypopituitarism were the sole presenting manifestations of metastatic spread. Pituitary metastasis from mandibular SCC is exceptionally rare and may present as the earliest sign of distant disease.

## Introduction

Metastases to the pituitary gland are uncommon manifestations of systemic malignancy. Autopsy studies estimate microscopic pituitary metastases in 1%–3% of patients with cancer, while clinically apparent lesions account for <1% of intracranial secondary tumors [1–3]. Breast and lung carcinomas are the most common sources. Pituitary metastases from squamous cell carcinoma (SCC) of the oral cavity are particularly uncommon, as these tumors typically spread locally and regionally before distant dissemination [1, 4, 5].

In head and neck carcinoma, distant metastases most frequently involve the lungs, bone, and liver, occurring in ~10% of patients [6]. Brain metastases are reported in only 2%–8% of cases and typically involve the cerebral hemispheres or cerebellum [6, 7]. Pituitary involvement is exceedingly rare, with isolated reports describing pituitary metastases from tonsillar or glottic SCC and occasional cases from papillary thyroid carcinoma [8–12]. Merchant *et al*. described the first isolated pituitary fossa metastasis from tonsillar SCC, followed by additional reports of endoscopic resection and isolated intracranial disease after curative treatment [8–10].

Clinical manifestations of pituitary metastases are nonspecific and include diabetes insipidus, cranial neuropathies, hypopituitarism, and headache, with diabetes insipidus reported in 30%–70% of symptomatic patients [1, 2, 13]. In over half of cases, pituitary-related symptoms represent the initial presentation of malignancy [13]. Imaging features may suggest metastatic disease but are not diagnostic [1–3].

We report a rare case of pituitary metastasis from mandibular SCC in which pituitary dysfunction was the first clinical indication of distant spread, underscoring the need for heightened suspicion in patients with oral cavity cancer presenting with new visual, cranial nerve, or endocrine symptoms.

## Case presentation

### Initial presentation and diagnosis

**Figure 1:**
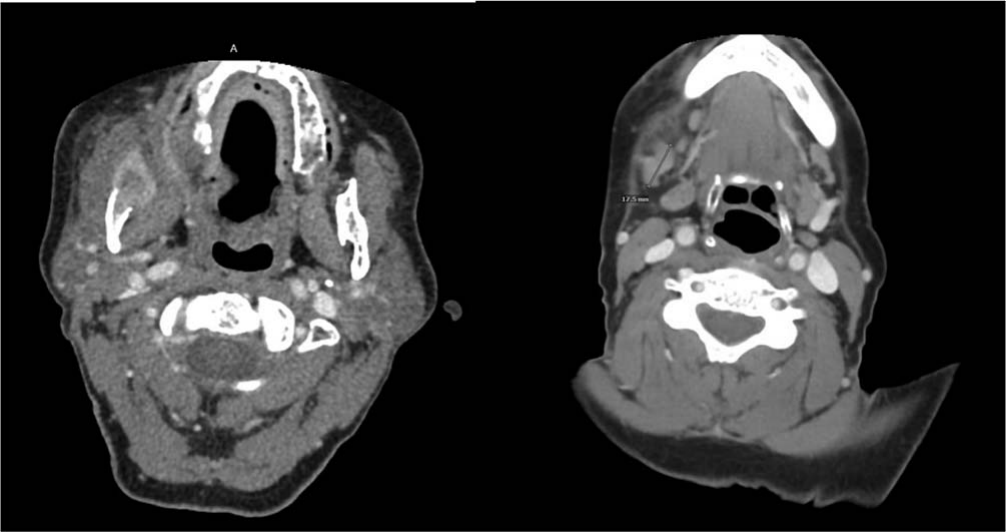
CT neck with contrast, near time of initial diagnosis: Infiltrative, erosive 3.8 × 2.5 × 4.1 cm soft tissue mass is seen involving the right hemimandible with locoregional soft tissue infiltration. Enlarged right level 1B lymph nodes are seen measuring up to 1.1 × 1.6 cm which may be reactive in nature or reflect nodal metastases.

**Figure 2:**
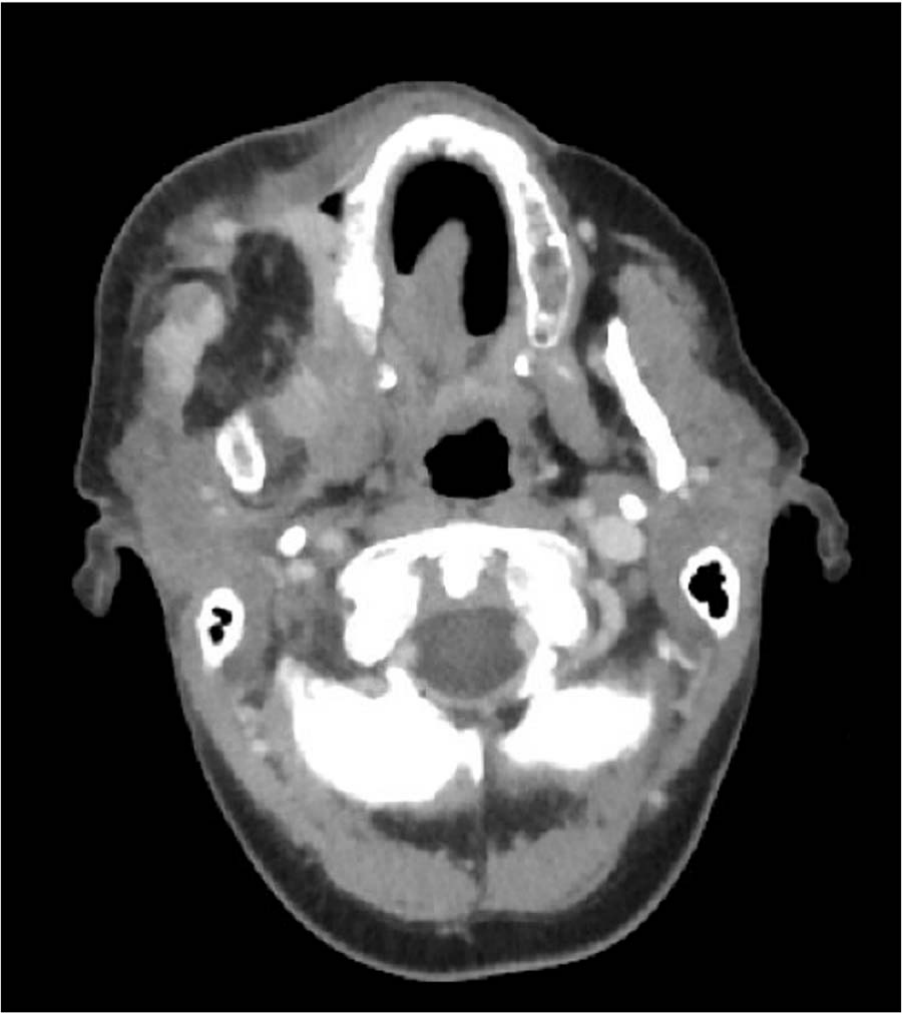
CT neck with contrast 2 months post-surgery: Focus of enhancement deep to resection cavity within pterygoid musculature measuring 1.3 × 1.1 cm with associated radiotracer uptake.

A 47-year-old female with a history of gastroesophageal reflux disease status post gastric bypass and prior hysterectomy presented with 2–3 weeks of right-sided facial swelling. Neck imaging revealed a 3.8 × 2.5 × 4.1 cm infiltrative, erosive mass involving the right hemimandible with associated soft tissue invasion and enlarged right level 1B lymph nodes up to 1.6 cm. Biopsy of the right posterior mandible confirmed well-differentiated SCC. Positron emission tomography/computed tomography (CT) demonstrated hypermetabolic activity in the mandibular lesion and right level 1B and 2 lymph nodes without evidence of distant metastasis (Figs 1 and 2).

### Surgical management

 The patient underwent surgical resection via segmental mandibulectomy and right-sided neck dissection with reconstruction using a right scapular free flap. Pathologic evaluation of the resected specimen revealed a spindle cell variant of SCC, with a primary tumor measuring 7 cm and invading through the cortical bone of the mandible to a depth of >10 mm. Positive surgical margins, extensive lymphovascular invasion (LVI), perineural invasion (PNI), and extranodal extension (ENE) were noted. Of 44 excised lymph nodes, 4 were positive for metastatic carcinoma, with the largest measuring 2.5 cm. Final staging was pT4aN3bM0.

### Adjuvant therapy

Adjuvant chemoradiation was initiated within 2 months, consisting of 6996 cGy in 33 fractions with concurrent weekly cisplatin. Treatment was complicated by severe mucositis, a perforated marginal gastric ulcer requiring surgical repair and gastrostomy tube placement, hemoptysis, and a segmental pulmonary embolism managed with apixaban.

### Pituitary metastasis and rapid decline

**Figure 3:**
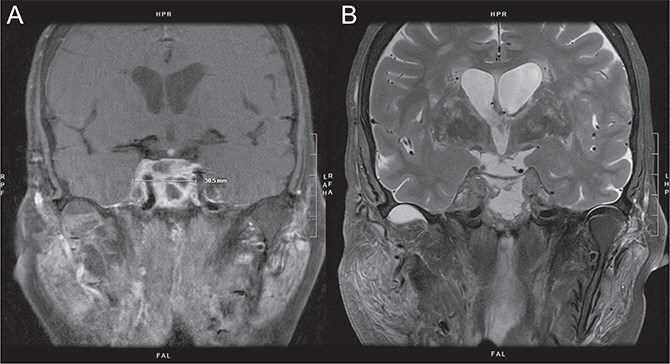
(A and B) MRI brain with contrast, 7 months after initial diagnosis: Heterogenous enhancing sellar mass with involvement of bilateral cavernous sinuses ~23 × 31 × 29 mm.

Approximately 5 months postoperatively, the patient developed progressive headaches, retro-orbital pain, facial numbness, and right eye ptosis. Magnetic resonance imaging (MRI) demonstrated a 2 × 2 × 2.6 cm heterogeneously enhancing sellar mass with bilateral cavernous sinus involvement (Fig. 3A and B). Neurologic examination revealed cranial nerve III palsy. Endoscopic endonasal biopsy confirmed metastatic SCC consistent with the mandibular primary.

Within weeks, the patient experienced rapid neurologic deterioration with altered mental status and meningismus. MRI showed leptomeningeal enhancement concerning for leptomeningeal carcinomatosis. Despite palliative ventriculoperitoneal shunt placement, the patient declined rapidly and died 6 months after diagnosis.

## Discussion

Pituitary metastases are rare in SCC, with most cases arising from breast or lung primaries. This case is distinctive in that pituitary involvement represented the first clinical manifestation of distant spread in mandibular SCC, without prior evidence of metastasis. Initial symptoms included headache, cranial nerve deficits, and eye pain. To our knowledge, this represents one of the few reported pituitary metastases from head and neck SCC (HNSCC) and the first arising from a mandibular primary.

Pituitary metastases are a recognized but uncommon manifestation of systemic malignancy, most frequently arising from breast and lung primaries. However, this case provides several novel insights beyond the presence of pituitary involvement alone. Notably, this represents an instance of mandibular SCC presenting with isolated pituitary metastasis as the first indication of distant disease, without evidence of systemic dissemination on staging imaging. This pattern deviates from the typical metastatic paths of head and neck SCC, which more often involves the lungs, bone, or liver and usually occurs in the context of advanced, disseminated disease.

An additional important consideration highlighted by this case is the diagnostic challenge posed by sellar lesions in oncology patients. The imaging appearance and clinical presentation of pituitary metastases can overlap significantly with primary pituitary adenomas or other benign sellar pathologies. In this patient, the combination of cranial nerve III palsy, retro-orbital pain, and a sellar mass could plausibly have been attributed to a primary pituitary process. This underscores the importance of maintaining oncologic suspicion and pursuing early biopsy, particularly in patients with a history of head and neck malignancy.

Although pituitary metastases account for only a small proportion of surgically treated sellar lesions, autopsy studies suggest subclinical involvement is more common, indicating that many cases go undiagnosed during life [1, 2, 5]. Breast and lung cancers predominate as primary sources [1, 5]. In contrast, HNSCC typically spreads locoregionally via cervical lymphatics, with distant metastases most often involving the lung, bone, and liver; intracranial spread is rare and usually reflects advanced disease [6, 7, 10, 14].

Morita *et al*. reported that over half of patients with symptomatic pituitary metastases presented with pituitary-related complaints prior to cancer diagnosis [13]. However, reported intracranial metastases in HNSCC generally occur in the setting of widespread disease [6, 7, 15]. This case expands current understanding by demonstrating that pituitary metastasis may signal early hematogenous dissemination in mandibular SCC, even in the absence of other detectable metastases.

Clinicians should maintain suspicion for pituitary metastasis in oral cavity SCC patients presenting with new visual, cranial nerve, or endocrine symptoms, prompting pituitary MRI and endocrine evaluation [2, 3, 8]. Management is individualized and palliative, with surgery and radiotherapy offering symptomatic relief, though prognosis remains poor and dependent on systemic disease burden [1–3, 11, 13]. In summary, this case challenges the assumption that distant metastases in oral cavity SCC follow predictable patterns and demonstrates that clinically occult, atypical metastatic spread may first present through pituitary dysfunction. In patients with head and neck SCC, new visual, cranial nerve, or endocrine symptoms should prompt early pituitary imaging and consideration of metastatic disease, even without known systemic spread.

## Conclusion

This case represents one of the first reports of pituitary metastasis from mandibular SCC as the initial manifestation of distant spread. Clinicians should maintain a high index of suspicion for pituitary involvement in patients with advanced SCC presenting with neurologic symptoms. Early diagnosis and intervention may guide management strategies, although prognosis remains poor in cases of leptomeningeal carcinomatosis.
